# Current status of the adjustable transobturator male system (ATOMS^TM^) for male stress urinary incontinence

**DOI:** 10.3389/fsurg.2024.1377788

**Published:** 2024-03-19

**Authors:** Patrick Juliebø-Jones, Ingunn Roth, Lazaros Tzelves, Karin M. Hjelle, Christian Arvei Moen, Francesco Esperto, Bhaskar K. Somani, Christian Beisland

**Affiliations:** ^1^ Department of Urology, Haukeland University Hospital, Bergen, Norway; ^2^ Department of Clinical Medicine, University of Bergen, Bergen, Norway; ^3^ Second Department of Urology, National and Kapodistrian University of Athens, Sismanogleio General Hospital, Athens, Greece; ^4^ Department of Urology, Campus Bio-Medico University, Rome, Italy; ^5^ Department of Urology, University Hospital Southampton, Southampton, United Kingdom

**Keywords:** adjustable transobturator male system, artificial urinary sphincter, male stress urinary incontinence, post prostatectomy incontinence, ATOMS system

## Abstract

Male stress urinary incontinence is a debilitating condition, which can occur after prostate surgery. In persistent cases, surgery is indicated and a number of options are available. This includes one of the male slings, Adjustable transobturator male system (ATOMS^TM^, A.M.I, Austria). There are now an increasing number of studies published. This review provides an overview of the current status of this implant device including technical considerations, surgical outcomes and potential advantages and disadvantages compared to alternatives such as the artificial urinary sphincter.

## Introduction

Male stress urinary incontinence (SUI) is one of the major long term adverse effects that can occur following prostate surgery and in particular, radical prostatectomy (RP). According to a recent analysis of the SEER (Surveillance, Epidemiology and End Results) database, 6% of patients who have undergone RP, will later go on to have incontinence surgery (1). Risk factors include larger prostate size, membranous urethral length and age (2, 3). The resulting impact on a patient´s quality of life can be considerable. As for many other patient groups who suffer UI, it can lead to embarrassment and deep restrictions upon a persońs activities of daily living (4). This sense of embarrassment can lead to delays in seeking formal treatment (5). Once the indications for incontinence surgery have been fulfilled, a range of potential surgeries are available. Local expertise, surgeon preference and availability impact the range of options a patient will be offered. Other elements to consider include the symptom severity, manual dexterity as well as previous radiotherapy (6). While use of bulking agents is the least invasive option, success rates are low and it is no longer recommended by the European Association of Urology guidelines (7). Rather, implantable devices form the mainstay of surgery for male SUI. This treatment can be broadly categorised into two groups: the artificial urinary sphincter (AUS) and male slings. The latter can be further divided into devices that are referred to as adjustable and those that are fixed. Regarding the former, several different such slings are available including ATOMS^TM^ (A.M.I, Austria). First described in a cadaveric setting in 2005, it was developed and in use for clinical purposes since 2008 (8, 9). Since then, a gradual increase in original studies have been published reporting outcomes associated with the device including for patients with a broader selection criteria. It is usually compared against the AMS 800^TM^, which since it has been commercially available since 1983, is the intervention for which most evidence is available and as a result, it has long been the reference treatment (10). However, the advances recorded with use of ATOMS^TM^ have generated debate in terms of the role it should play for male SUI post prostate surgery including when and if it can be a preferred choice over the AMS 800^TM^.

Our aim was to review the available literature and provide an update on its current status including technical considerations, surgical outcomes and the guideline perspectives.

## Methods

A comprehensive but non-systematic search of the literature was performed to identify studies on ATOMS^TM^ that were available over the past 15 years since it was first described. Only those published in the English language were considered. Bibliographic databases used included Medline and Google Scholar. The following key topics were identified: Technical considerations, Short term outcomes, Long term outcomes, Complication burden, Re-do surgery, Previous radiotherapy, ATOMs for severe male SUI, Advantages/disadvantages, Recommendations from international guidelines, Challenges and Future directions.

### History and technical considerations

The initial clinical experiences to be published appeared a few years after its development. The Austrian study by Seweryn et al., which included 38 patients since 2009 was among the first (11). The authors reported continence success (defined as maximum one pad per 24 hours) at 60.5%. Since that report, the device has undergone modifications, most notably in 2013 with a silicone rather than titanium port cover and after 2014, this could be placed in the scrotum with the port pre-attached. This element along with its mesh arms and silicone cushion form key characteristics that distinguish it from adjustable male sling alternatives such as the Remeex system (Neomedic, Spain), consisting of a suprapubic pressure adjusting device (“varitensor”) connected via two traction threads to a suburethral prosthesis made of polypropylene and the Argus sling (Promedon, Argentina) (6, 12). The latter features a silicone cushion pad to compress the bulbar urethra, cone shaped columns on either side and “washers” to secure tension (13).

The switch to a silicone covered port was driven by early reports of device explantation as a result of reported titanium intolerance (14). The transition away from the inguinal port placement has reduced the total number of incisions required from two down to only one. The 3rd generation port is also smaller. In a multi-centre study from Germany and Spain, port related complications were 19.2% with the first device but 6.5% with the 3rd generation model (15).

In a recent series by Giammò et al., the mean operative time was 51 min, and most studies report similar results that total operative times under 60 min (16). Hospital discharge is usually planned for within 24 h and adjustments can be made to the port in the outpatient setting. In a recent systematic review, the mean number of fillings required was 2.4 (17).

### Symptom improvement

In a 2019 meta-analysis of pooled data from 1,393 patients (20 studies), 90% were found to have symptom improvement at follow up (18). Sample sizes of the included studies ranged from 13 to 287 patients. Seven of the included studies reported patient satisfaction and rates ranged from 61.8% to 100%. Interestingly, in the study with a satisfaction rate of 61.8%, 39% of the sample had undergone previous radiotherapy and the explantation rate was 31.5% (19). In contrast, in the study recording 100% satisfaction, only 7.7% had received previous radiotherapy and the explantation rate was zero (20). More research is needed to determine what affects long term patient satisfaction.

Pooling data on dryness rates is difficult given the varying definitions for this parameter. In a retrospective study of 155 patients by Angulo et al. published in 2020, which had a mean follow up of 60 months, 72.1% achieved a dry status as defined by no pads or one security pad (21). Friedl et al. reported on the impact of ATOMS^TM^ on sexual function (22). Erectile function scores were improved at six months follow up and 38% of the sample started to have intercourse again after having stopped previously. Dual implantation of penile prosthesis and ATOMS^TM^ has been reported. To date, only data on simultaneous AUS placement at the time of surgical repair of refractory bladder neck contracture has been reported (23–25).

### Complications

Muhlstadt et al. reported the overall complication rate to be 27.3% in their series of 187 patients. The authors found previous radiotherapy as well as previous urethral surgery to be significant predictors of a post operative complication (15). The learning curve associated with ATOMS was also studied and found the rate of complication to fall from 44% to 21.1% after 25 cases were performed. Angulo et al. reported an explantation rate of 8.5% in their multi-centre study of 902 patients (26). The two commonest indications for removal were persistent incontinence and port erosion. Explantation rates do vary, with reports of as high as 19% previously recorded (27). Possible reasons for such wide variations include different follow up lengths and the proportion of patients with radiotherapy. Giammo et al. reported the survival of the ATOMS^TM^ device to be 97% at 12 months and 89.9% at 60 months (28). As with all prosthetic devices, infection can be a major complication and rates between 2.7%–6.2% have been reported (8). Predictive nomograms have become increasingly popular across many areas of urology and tools are now available in the setting of ATOMS^TM^ (29, 30). This includes the tool developed by Dorado et al., which serves to predict risk of failure (30). Variables included in that tool are Male Stress Incontinence Grading Scale (MSIGS), 24-h pad test and history of radiotherapy.

### Guidelines perspective

While ATOMS^TM^ surgery is covered in the latest EAU guideline, no formal recommendations regarding any of the adjustable male slings are given as the panel determined the current body of literature to be still lacking (31). Fixed male slings do however receive a recommendation. Here it is stressed that the role of such fixed devices should be limited to the setting of men with mild to moderate incontinence. Again however, it is underscored that the evidence is also limited for this intervention type. The American Urological Association guidelines do not make any specific comment regarding the ATOMS^TM^ device (2). Male slings as a group are discussed with a similar recommendation that they are avoided in the setting of severe incontinence. These positions shared by the EAU and AUA guidelines are very similar to those from the Urological Society of India, Canadian Urological Association (CUA) and the International Consultation on Incontinence (ICI) (32–34). Of note, some of these guidelines are not updated yearly. The CUA document was disseminated in 2012, which was before the last generation of ATOMS^TM^ was released and there have been multiple original studies published since then (32). Bhatt et al. evaluated all five of these guidelines on the topic of post prostatectomy incontinence using the Appraisal of Guidelines for Resarch and Evaluation II (AGREE II) tool on domains such as scope, clarity and applicability (35). The authors concluded the AUA guidelines to score highest.

### Advantages and disadvantages compared to AUS

There are several advantages that can be found with the ATOMS^TM^ device. Firstly, and in contrast to fixed slings such as the AdVance™ and the AUS, adjustments can be made without the need to return to surgery and the scrotal port placement allows for ease of access when doing so. Also, if there is a complication that is localised to the port only, the port can be removed in isolation. While this means that further adjustments cannot be made, it avoids the complete explantation of the device. ATOMS^TM^ is also associated with a shorter operative time compared to AUS. In a propensity-score-matched analysis comparing the two devices, the mean operative time associated with ATOMS^TM^ was significantly shorter (56 vs. 100 min, *p* < 0.001) (36). In contrast to AUS where satisfactory manual dexterity and cognition is required, patients are not required to manipulate the ATOMS^TM^ device themselves. While simpler methods for activation of the AMS 800 have been proposed, these are not yet in clinical use. Even if patients have normal cognition and dexterity at the time of surgery, if they later suffer an acute medical event such as a stroke that impairs their upper motor function and/or their cognitive status, this can pose obvious problems for those with an AUS *in situ*. From a practical perspective, patients with AUS also require greater caution when performing a subsequent cystoscopy as well as the need for a urologist to attend the operating theatre to deactivate the AUS device if undergoing surgery by another specialty when catheterization is being performed. From an anatomical perspective, the non-circumferential design reduces the risk of urethral atrophy and erosion. Infection rates are also lower when compared to AUS as well as Argus and Remeex. There is a potential cost advantage too, with Constable et al. reporting costs associated with ATOMS^TM^ procedure to be £6,000 compared to £9,000 with the AUS (37).

However, as raised by international guidelines, the levels of evidence supporting the role of ATOMS^TM^ is more limited compared to AUS. This is perhaps the biggest disadvantage. How these abovementioned advantages translate overall is thus yet to be fully determined.

While the body of original studies for ATOMS^TM^ does exceed 20, many are single centre and retrospective in nature and to date, there have been no randomised studies, which have placed ATOMS^TM^ head-to-head against AUS. The MASTER trial did compare male slings with AUS but most of the slings included in that non inferiority trial were the fixed type and a full breakdown is not given (38). The authors found no differences in SUI burdens at follow up. However, secondary outcomes such as complication rates did favour AUS. The proposed advantages of the AUS are its feasibility in patients with previous radiotherapy and those with severe SUI.

### Challenges

Beyond the abovementioned lack of studies in comparison to other incontinence devices, other challenges exist. For example, the lack of standardised reporting as well as lack of consensus regarding reporting of SUI. Some author groups prefer to use pad count while others choose pad weight. Furthermore, for each one of these, consensus is lacking regarding how to how to grade severity. This makes comparisons between studies more difficult. Another area that appears to lack standardisation is reporting of port removal/total explantation. For example, some groups report this as a complication but others consider it a late treatment failure. Unless a reader studies the results very carefully and is aware of this, one can easily misinterpret the complication burdens across different studies.

Heterogeneity in other forms is also common among studies. For example, populations with both RP and benign prostate surgery patients and some having had radiotherapy. Furthermore, radiotherapy type (e.g., adjuvant vs. salvage) is not routinely specified in these studies. Studies reporting their experiences with ATOMS^TM^ over several years will usually include patients have had different generations of ATOMS^TM^ devices. This can introduce further bias.

## Conclusions

With over a decade of published results associated with ATOMS^TM^ now available, this adjustable sling device has positioned itself as an effective surgical option. It offers strengths that can complement the longer established AUS. Further studies will allow for optimal selection criteria to be further defined and its recommended role in international guidelines to be delineated. This includes the role of ATOMS^TM^ in the setting of previous radiation as well as severe incontinence.
